# Wealth Status, Mid Upper Arm Circumference (MUAC) and Antenatal Care (ANC) Are Determinants for Low Birth Weight in Kersa, Ethiopia

**DOI:** 10.1371/journal.pone.0039957

**Published:** 2012-06-29

**Authors:** Nega Assefa, Yemane Berhane, Alemayehu Worku

**Affiliations:** 1 College of Health Science, Haramaya University, Harar, Ethiopia; 2 Addis Continental Institute of Public Health, Addis Ababa, Ethiopia; 3 School of Public Health, Addis Ababa University, Addis Ababa, Ethiopia; University of Vermont, United States of America

## Abstract

**Background:**

Low Birth Weight (LBW) is one of the major risk factor for death in early life. However, little is known about predictors of LBW in sub-Saharan Africa. Therefore, the aim of this study was to measure the incidence and determinants of LBW in a rural population of Ethiopia.

**Methods:**

An observational cohort study on pregnant women was conducted from December 2009 to November 2010. During the study period 1295 live birth were registered and the weights of 956 children were measured within 24 hours after birth. Socio-demographic, economic, maternal and organizational factors were considered as a predicators of LBW, defined as birth weight below 2500g. Logistic regression was used to analyze the data, odds ratio (OR) and confidence intervals (CI) are reported.

**Result:**

The incidence of LBW was 28.3%. It is significantly associated with poverty [OR 2.1; 95% CI: 1.42, 3.05], maternal Mid Upper Arm Circumference (MUAC) less than 23 cm [OR 1.6; 95% CI: 1.19, 2.19], not attending ANC [OR 1.6; 95% CI: 1.12, 2.28], mother’s experience of physical violence during pregnancy [OR 1.7; 95% CI: 1.12, 2.48], and longer time to walk to health facility [OR 1.6; 95% CI: 1.11, 2.40].

**Conclusion and Recommendation:**

The incidence of LBW was high in Kersa. Babies born to women who were poor, undernourished, experienced physical violence during pregnancy and who had poor access to health services were more likely to be LBW in this part of the country.

In this largely poor community where ANC coverage is low, to reduce the incidence of LBW, it is essential to improve access for maternal health care. The involvement of husbands and the community at large to seek collective action on LBW is essential.

## Introduction

Low Birth Weight (LBW) is one of the risk factors for child morbidity and mortality [1], [2]. A child’s birth weight is an important indicator of the child’s vulnerability to the risk of childhood illnesses and the chances of survival. Children whose birth weight is less than 2.5 kilograms, are considered to have a higher than average risk of early childhood death [3], [4].

Though its extent varies from place to place, LBW is a common phenomenon in many parts of the world. For example, in North America, its prevalence is less than 6% [5], studies in Thailand, Pakistan and Iran showed its prevalence ranges from 8–15% of the annual births [6]–[9].

In Harare teaching hospital, Zimbabwe, per 1000 live births the annual incidence of LBW was 199 [10]. In Ethiopia, studies conducted in the year 2000 and 2004 in Addis Ababa and around Jimma rural communities showed that the prevalence of LBW was 8.8% and 10%, respectively [11], [12]. According to the Ethiopian Demographic and Health Survey (DHS) report, 23% of babies born in the rural setting were LBW. The same report showed the prevalence of LBW for rural Oromia region was 20.5% [3].

LBW is routinely associated with several maternal factors such as younger and older age, rural residence, low family economic status and illiteracy [8]–[10], [13]–[16]. Women who had low Mid Upper Arm Circumference (MUAC) measure and low Body Mass Index (BMI) are more likely to give birth to LBW babies than their counterparts [17], [18].

Women, who have three or more children, who are not living with the father of the new born, who have multiple gestations, who have unintended pregnancy and who have a history of delivering LBW have an increased risk of delivering LBW baby [6], [13], [14], [19], [20].

Similarly pregnancy co-morbidities such as existence of hypertension, anemia, and poor maternal weight gain during the index pregnancy increases the risk of having LBW baby [8], [10]. The number of ANC visits and parity are significantly associated with LBW. Attending less than four ANC visits doubles the risk of LBW compared to those visiting four times [17], [18].

In Ethiopia literature on LBW is scarce. The study conducted in Addis Ababa used retrospective data from a tertiary teaching hospital. Clients who visited such hospitals are usually referred by other hospitals. Even then it is only those who can afford to stay in Addis Ababa accesses the service. Therefore, they are different from those who give birth in the usual clinical setting. The study conducted around Jimma used weight measured up to the 7^th^ day after delivery. The measurement obtained on the 7^th^ day is different from the measurement that would be obtained on date of birth. Therefore, they are not true reflection of birth weight [11], [12].

On the other hand, the DHS results were based on the verbal report of the baby’s weight, and active baby weighting was not done. In addition, DHS looked for incidents of LBW for babies born in the last five years preceding the survey [3]. Reporting correctly retrospective information of such kind depends on how far the memory goes and the attention given by the mother and the family to remember the figure, which in most cases is unlikely.

Thus, it was found useful to conduct a study in rural settings where 84% of the Ethiopian population lives. The aim of this study was to identify the incidence and determinants of LBW in the rural community.

Identifying determinates of LBW and preventing them helps in reducing early childhood morbidity and mortality resulting from LBW. Therefore, addressing factors affecting the birth weight of babies will contribute to the achievement of the Millennium Development Goal (MDG) number 4. The knowledge gained from this study can be applied to the other parts of the country and beyond with similar characteristics.

## Methods

The study was conducted in Kersa Demographic Surveillance and Health Research Center (KDS-HRC) field site, Ethiopia from December 2009 to November 2010. The study site has two urban (Kersa and Weter towns) and 10 rural Kebeles (smallest administrative unit in Ethiopia, with an average population of 5000). According to the first census in 2007, the study site has 48, 192 inhabitants in 10, 256 households.

The health service in the district is provided by six health centers, 28 health posts and health extension workers posted in all the Kebeles. According to the health office in the district, the health coverage in 2011 is 80% [21]–[23].

The study was approved by the Ethiopian National Ethical Clearance Board. The mothers of the babies enrolled in the study were informed about the study, the nature of the study, its objectives, the expected outcomes, and the benefits and risks associated with the study. A written informed consent was obtained from the mothers.

Participants of the study were part of the pregnancy surveillance initiated in KDS-HRC [24]. Pregnant women were followed monthly and as necessary on weekly bases. On the day of deliveries village informants notified the data collectors about the event. The data collectors took weights, head and chest circumferences measurements within 24 hours. The measurements were made using portable standard measuring devices.

The newborns were weighted naked or in minimal clothing using a *Salter’s hang up scale* to the nearest 100 g following standard techniques [25]. Chest Circumferences were obtained at the level of the xipho-sternum and below the inferior angle of the scapula during quiet respiration, while the head Circumferences were measured by passing the tape between the supra orbital ridges and the maximum occipital prominence.

In addition, using a questionnaire adapted from relevant research reports [3], [26], data were collected by the data collectors, who were hired and trained for the pregnancy surveillance.

Data were entered using Epi data version 2.2 and analyzed using STATA software version 11 [27], [28]. The proportions of the independent variables were calculated against LBW. Unadjusted Odds Ratio (OR) was calculated and multi collinearity was tested. Maternal age, maternal education, family economic status, parity, MUAC, fertility desire, antenatal care, violence, travel time to the nearest health facility were the independent variables.

Birth weights of babies less than 2500g was labeled as LBW and 2500g and above was considered normal weight [29]–[31]. Further classification of birth weight into very LBW and LBW was not done.

During analysis, age was grouped using 10 year interval; maternal education was grouped as illiterate for those who can’t read and write and all others are labeled as literate. The ten rural Kebeles are labeled as rural and the two small towns (Kersa and Weter) are labeled as rural towns.

To determine the economic status of families, wealth index was used. The wealth distribution was generated by applying principal components analysis to 33 household variables [31]–[33]. Then it was grouped into three; better off, middle and poor. Parity other than the index birth was asked and later during analysis it was regrouped as 0 for primipara, 1–4, 5–6 and 7+ deliveries.

Travel time to the nearest health facility was used to assess access to health care and it was based on the women’s estimate. Later during analysis, the reports were grouped into less than 40, 40–79 and 80+ minutes. The grouping was made based on local people’s reference that “a woman can walk an estimated distance of 5 km in 40 minutes.”

To proxy maternal nutritional status MUAC was used. A MUAC of less than 23cm was considered to be a sign of poor nutrition. MUAC doesn’t vary much during pregnancy and is therefore an appropriate measure of nutritional status than BMI or weight [34], [35]. Violence was asked using adapted World Health Organization violence questions [26]. Reports of either of slapped, shoved, dragged, threated with gun or knife was recorded as Physical violence. And manners of unconsented sex or sexual advances were recorded as sexual violence. If the women did not report experience of such violent acts during the index pregnancy, it was recorded as no violence.

Fertility desire was recorded based the report of the women. All mistimed and unwanted pregnancies were labeled as unintended and otherwise intended. ANC attendance was recorded using woman’s report or report plus ANC card. Women who reported to visit at least once were labeled as ANC attended and those reported not visiting health institution for ANC attendance were labeled as not attended.

Before entering the variables in to regression models, missing birth weight was analyzed. Missing birth weight was 26.2%. It was significantly associated with women from poor family, women with MUAC of 23+ cm; experience of physical violence during pregnancy and travel time to the nearest health facility less than 40 minutes. This suggests missed observations were significantly different from those allowed their babies to be measured. Therefore, to curb the problem of missing to the outcome variable (Birth Weight) sample weighting was used for those who allowed their children to be weighted. The purpose of sample weighting was to improve representativeness of the sample in terms of characteristics of the study population [36]. After doing this, all the remaining analyses were done based on weighting.

Finally data were analyzed using four separate regression models. The first three models were used to see the relative effect of variables each other in block before entering them in the final model. In model one socio demographic and economic variable were entered. In model two parity and maternal nutritional status were entered. In model three variables relating to the index pregnancy were entered. In the final model all variables were entered together. Statistical level of significance was set at alpha 5%.

## Results

A total of 1295 live births were registered and of these the weights of only 956 babies were measured. This gives a retention rate of 73.8%. The remaining babies were not weighted because the mothers did not let their babies weighted for cultural and traditional reasons. LBW was detected among 271 babies (28.3%; CI 25.5, 31.2).

More LBWs were observed among women whose ages were less than 20 and more than 40, who were illiterate, who were rural resident and who were poorer than their counterparts. LBW was significantly associated with rural residence and poor wealth status (p<0.05 for both) (Table 1).

**Table 1:** Basic Characteristics of study subjects weighted unadjusted Odds Ratio, Kersa, 2010.

Characteristics		Totaln = 956	<2500g%	2500+g%	Crude OR	CI		p = value
Age	<20 Years	66	30.3	69.7	1.0			
	20–29 Years	422	26.3	73.7	0.8	0.47	1.47	0.526
	30–39 Years	442	29.9	70.1	1.0	0.57	1.76	0.997
	40+ Years	26	30.8	69.2	1.0	0.37	2.65	0.975
								
Educational level	Literate	144	25.0	75.0	1.0			
	Illiterate	812	28.9	71.1	1.2	0.80	1.82	0.359
								
Residence	Rural town	88	13.6	86.4	1.0			
	Rural	868	29.8	70.2	2.7	1.46	5.12	0.002
								
Wealth status	Better off	309	21.4	78.6	1.0			
	Middle	329	26.8	73.3	1.3	0.93	1.92	0.123
	Poor	318	36.8	63.2	2.1	1.46	2.98	0.000
								
Parity	First time	111	31.5	68.5	1.0			
	1–4 birth	559	27.1	72.9	0.8	0.52	1.26	0.342
	5–6 birth	164	26.8	73.2	0.8	0.47	1.35	0.390
	7+ birth	122	33.6	66.4	1.1	0.62	1.88	0.786
								
MUAC	23+ cm	504	24.6	75.4	1.0			
	<23 cm	452	32.6	67.4	1.5	1.14	2.01	0.004
								
Fertility desire	Intended	676	29.6	70.4	1.0			
	Unintended	280	25.4	74.6	0.8	0.58	1.10	0.171
								
Antenatal care	Attended 1+	281	20.6	79.4	1.0			
	Not attended	675	31.6	68.4	1.8	1.28	2.49	0.001
								
Violence during index pregnancy	No violence	765	26.9	73.1	1.0			
	Any physical	148	38.5	61.5	1.8	1.22	2.54	0.003
	Sexual only	43	18.6	81.4	0.6	0.28	1.33	0.211
								
Time to walk to health facility	<40 min	274	20.4	79.6	1.0			
	40–79 min	621	32.5	67.5	1.8	1.30	2.56	0.001
	80+ min	61	21.3	78.7	1.0	0.52	2.03	0.949

Odds Ratio/OR is calculated using logistic regression; Low Birth Weight (<2500g) coded as = 1

P values are based on results from logistic regression

Compared to parity of 1–4 and 5–6; primipara and parity of 7+ had more LBW babies. Women who had MUAC of less than 23 cm had more LBW babies than those who had MUAC of 23 cm and more. Unadjusted OR also showed a statistically significant association between LBW and MUAC of less than 23 cm (Table 1).

More LBW babies were observed among women who never followed antenatal care than those who attended one or more times. Experience of physical violence during the index pregnancy increased the risk of having LBW, compared to those who experience sexual violence or no violence. Women who reported estimated time to walk to the nearest health facility 40–79 minutes had more LBW babies than those who reported less than 40 minute and more than 80 minutes. Significant associations between LBW and antenatal follow-up, physical violence and 40–79 minutes to walk to health facility were detected (Table 1).

In the first block logistic regression model, LBW was significantly high among rural residence and poor, (OR 2.2; 95% CI 1.16, 4.34) and (OR 1.9; 95% CI 1.29, 2.68), respectively. In the second model, LBW was significantly associated with MUAC less than 23 cm (OR 1.5, 95% CI 1.13, 2.0).In the third model, LBW was significantly associated with not attending ANC and experience of physical violence during the index pregnancy, (OR 1.8, 95% CI 1.3, 2.54) and (OR 1.8, 95% CI 1.20, 2.57), respectively (Table 2).

In the final mode, LBW was significantly associated to the poor (OR 2.1,95% CI 1.42, 3.05), MUAC less than 23 cm (OR 1.6, 95% CI 1.19, 2.19), not attending ANC (OR 1.6, 95% CI 1.12, 2.28), experience of physical violence (OR 1.7, 1.12, 2.48) and time to walk to health facility 40–79 minutes (OR 1.6, 95% CI 1.11, 2.40) (Table 2).

**Table 2:** Logistic regression output of determinants of Low Birth Weights birth, Kersa, Eastern Ethiopia, 2010.

Characteristics		Model -I			Model –II			Model-III				Final Model- Weighted OR				
		OR	CI		OR	CI		OR	CI			OR		CI		
Age	<20 Years	1.0										1.0				
	20–29 Years	0.9	0.47	1.54								1.0	0.51		1.89	
	30–39 Years	0.9	0.51	1.69								0.9	0.47		1.87	
	40+ Years	1.0	0.34	2.73								1.0	0.33		3.31	
Educational level	Literate	1.0										1.0				
	Illiterate	0.9	0.60	1.47								0.9	0.58		1.46	
Residence	Rural town	1.0										1.0				
	Rural	2.2*	1.16	4.34								1.1	0.54		2.42	
Wealth status	Better off	1.0										1.0				
	Middle	1.2	1.29	2.68								1.4	0.96		2.10	
	Poor	1.9**	1.29	2.68								2.1***	1.42		3.05	
Parity	Never gave birth				1.0							1.0				
	1–4 birth				0.8	0.54	1.29					0.7	0.43		1.19	
	5–6 birth				0.8	0.49	1.41					0.7	0.36		1.26	
	7+ birth				1.1	0.65	1.93					1.1	0.56		2.14	
MUAC	23+ cm				1.0							1.0				
	<23 cm				1.5**	1.13	2.00					1.6**	1.19		2.19	
Fertility desire	Intended							1.0				1.0				
	Unintended							0.8	0.58		1.10	0.7	0.50		1.03	
ANC	Attended 1+							1.0				1.0				
	Not attended							1.8***	1.30		2.54	1.6**	1.12		2.28	
Violence duringindex pregnancy	No violence							1.0				1.0				
	Any physical							1.8**	1.20		2.57	1.7*	1.12		2.48	
	Sexual only							0.5	0.25		1.20	0.5	0.22		1.01	
Time to walk to health facility	<40 min											1.0				
	40–79 min											1.6*	1.12		2.40	
	80+ min											1.0	0.48		1.95	

* = p<0.05; ** = p<0.01; *** = p<0.001

## Discussion

In this study the incidence of LBW was 28. 3%. It was found that poor economy, MUAC less than 23 cm, not attending ANC, experience of physical violence and time to walk to health facility 40–79 minutes were strong predictors of LBW.

Obtaining birth weight data from the community setting within 24 hour is the major strength of the study. In many other researches, data on birth weight have not been collected within 24 hours due to the difficulty in acquiring information on delivery immediately after it happened [12]. We achieved this by using trained village informants who were residents of villages. In most Ethiopian cultures, the birth of a baby is welcomed by a special ritual and neighbors are expected to visit the mother. However, in some instances, even though the data collectors reached at the home of women, the families prevented the data collectors to weight the babies due to traditional beliefs. This had diminished the retention rate to 73.8%. To correct this, sample weighing was used and all the analysis takes into account the weights. If this was not done, during analysis incomplete observations were dropped. This had led to use a reduced sample size and gives a biased estimate. But with weighting, since one observation represents more than one observation, the missed data were compensated and relatively the confidence interval are tighter and results are not biased [36].

In assessing access to health care, time to walk to health facility was used. It was obtained by asking respondents to estimate the time they need to walk to the nearest health facility. In this study most of the respondents were illiterate, and their capacity to estimate time against distance may not be as accurate as the estimate that can be obtained from literate person.

In this study gestational age of the babies were not calculated. The main reason for this was majority of the women were unable to tell their last menstrual period. Therefore, it was not possible to control gestational age in the analysis.

The incidence of LBW in this study was higher than studies conducted in Zimbabwe and Ethiopia. The Harare teaching hospital study revealed an incidence of 199 per thousand live births, whereas the study conducted in Black Lion hospital of Ethiopia showed prevalence of 8.8% [10], [11]. These studies used tertiary hospital records; the health and other backgrounds of people using tertiary hospitals are different from the rural population. It is, therefore, likely that data from these hospitals suffered from selection bias [37]. The other study in Ethiopia which used a rural community setting had allowed measuring and recording baby’s weight up to 7^th^ day after birth, which means getting the correct birth weight is unlikely [12]. The other explanation for the increased incidence of LBW in this study could be due to poor maternal nutritional status as evidenced by high percentage of low MUAC measurements. The Ethiopian DHS estimate for LBW is closer to the estimate obtained in this study. However, it is hard to compare this finding with the DHS’s which is obtained by maternal report rather than measurement [3].

Poor wealth status was one of the strong predictors LBW. Similar findings were observed in other studies conducted in developing countries, including Sub-Sahara Africa. Failure to achieve adequate weight gain during pregnancy as a result of poor feeding pattern eventually affects the birth weight of the newborn. Reduction in the ability to seek medical care, travel for referrals and purchase of food/hygiene items are directly related to poverty, which indirectly affect the birth weight of the baby [8]–[10], [13], [19].

The nutritional status of women as proxy by MUAC was also a strong predictor of LBW. Other studies conducted in African countries also reached on similar conclusion [5], [13], [16], [19], [38]. Even though either acute or chronic maternal malnutrition has direct effect on the birth weight of a baby, acute maternal malnutrition has more pronounced effect.

Not attending ANC showed a significant association with LBW. Other studies had also identified similar finding [39]. In this study only 29% of the pregnant women had received ANC. In Ethiopia, according to the Federal Ministry of Health guideline on ANC, women who attended ANC should receive advice on balanced diet and the need for proper nutrition during pregnancy [18], [22], [23]. Health checkup for weight, height, gestational weight gain and health conditions are part of the routine procedure during antenatal care. As necessary, laboratory checks for hemoglobin and sexually transmitted illness including HIV should be done. Immediate intervention or referral is recommended if the women have positive finding in one of the tests and physical checkups. Provision of iron tablets is the other routine procedure for pregnant women [40].

Experience of physical violence during pregnancy was found to predict LBW. Violence creates stress and psychological disturbance that affects maternal nutritional balance [16], [41].

As time to walk to health facility increased more than forty minutes, the occurrence of low birth weight increased by 63%. In this study, more than 80 minute was not observed to increase low birth weight. This may be explained by the fewer sample size in that category as evidenced by wide confidence interval.

## Conclusions

The percentage of newborns delivered having LBW was extremely high in this study. The significant predictors of LBW were being poor, not attending ANC, MUAC less than 23 cm, experience of physical violence during the index pregnancy and longer time to walk to health facility. This is significant because LBW is highly associated with neonatal and infant morbidity and mortality.

Improving access to health care and improving the nutritional status of women particularity pregnant women would reduce the occurrence of LBW.

In Ethiopia, all rural Kebeles are staffed with health extension workers; these health workers are expected to provide house to house health promotion services. In this regard, they should emphasize providing ANC for the pregnant women. Through ANC, it is possible to identity a women who are at risk of having LBW baby. Giving necessary advice such as the need for balanced diet and provision of health promotion intervention such as provision of iron tables for the anemic can be easily facilitated.

In addition, empowering women, families and neighborhoods to work on their own health care such as peer discussion to use ANC, would reduce the number of babies borne having LBW. These activities would prevent not only the occurrence of LBW but also problems associated with LBW such as child morbidity and most importantly child mortality thereby helps the country achieve child MDG targets.
